# Conceptualizing Daily Dynamics of Social Connection in an Adult Lifespan Sample

**DOI:** 10.1093/geroni/igaf122.1685

**Published:** 2025-12-31

**Authors:** Jonathan Rush, Eric Cerino, Jennifer Piazza, David Almeida, Susan Charles

**Affiliations:** University of Victoria, Victoria, British Columbia, Canada; Northern Arizona University, Flagstaff, Arizona, United States; California State University, Fullerton, Fullerton, California, United States; The Pennsylvania State University, University Park, Pennsylvania, United States; University of California, Irvine, Irvine, California, United States

## Abstract

Social connection is central to many aspects of healthy aging, including increased emotional well-being and a reduced risk of mortality. Existing studies examining the link between social connection and health typically conceptualize social connection as a static, trait-like variable. Yet similar to other daily experiences, feeling socially connected fluctuates from day-to-day, and may provide additional insight into how social connection and health are linked. The present research examines three aspects of daily social connection: one structural (positive interactions) and two related to quality: feeling a sense of belonging; and responsivity (i.e., how sense of belonging varies in response to a positive interaction). Using eight days of daily diary data (N = 2,022), we examine the psychometric properties and utility of these three aspects of daily social connection. Each day, participants reported whether they had a positive interaction; their sense of belonging; and their closeness to others over the past 24 hours. Multilevel factor analyses revealed that daily feelings of belonging and closeness to others reflect a reliable unified social connection construct at both the within- and between-person levels (between-person factor loadings=.98, .87; within-person loadings=.67, .65, respectively; reliability=.92). Nearly 40% of the variability in daily social connection is within-person. Furthermore, these aspects of daily social connection demonstrated convergent validity to global indicators of social well-being. We discuss the value of utilizing daily social connection measures to effectively capture experiences of social connection within and between individuals, and how they can be used for future research examining the links between social connection and health.

